# Actuator Fault Detection for Unmanned Ground Vehicles Considering Friction Coefficients

**DOI:** 10.3390/s21227674

**Published:** 2021-11-18

**Authors:** Gyujin Na, Yongsoon Eun

**Affiliations:** Department of Information and Communication Engineering, DGIST, Daegu 42988, Korea; nagyujin@dgist.ac.kr

**Keywords:** unmanned ground vehicle, fault detection, unknown input observer, friction coefficient

## Abstract

This paper proposes an actuator fault detection method for unmanned ground vehicle (UGV) dynamics with four mecanum wheels. The actuator fault detection method is based on unknown input observers for linear parameter varying systems. The technical novelty of current work compared to similar work in the literature is that wheel frictions are directly taken into account in the dynamics of UGV, and unknown input observers are developed accordingly. Including the wheel friction, the vehicle dynamics are in the form of linear parameter varying systems. Friction estimation is also discussed in this work, and the effect of friction mismatch was quantitatively investigated by simulations. The effectiveness of proposed method was evaluated under various operation scenarios of the UGV.

## 1. Introduction

Control of unmanned ground vehicles (UGVs) has been an actively researched topic with the increased interest in autonomous vehicles [1,2]. The use of UGVs has been proven to be successful in missions such as reconnaissance, search, rescue, and delivery services [3]. In particular, they were sent to a dangerous environment for the purpose of monitoring unknown terrains [4]. They are expected to have infinite potential for supporting humans [5,6,7].

Among several UGVs, mecanum wheel vehicles have operation advantages in that they can move in any direction. Unlike a simple differential drive, a mecanum-wheeled drive has three degrees of freedom, which allows movement in all directions; i.e., it can move sideways or rotate around its own axis [8]. The mecanum wheel is based on a tireless wheel and the rollers have an axis of rotation at 45 degrees to the wheel plane and 45 degrees to the axle line [9]. The vehicles with mecanum wheels are being widely used for carrying heavy goods in an industrial environment [10,11].

Besides control of UGV, actuator fault detection methods have received much attention. Reference [11], for instance, presented a fault detection method for UGV with four mecanum wheels. The authors developed an actuator fault detection method based on unknown input observer (UIO), and it isolates faulty actuators targeting the friction-free UGV model. However, since UIO is a model based state estimation method, designing a fault detector without considering the effect of friction degrades fault detection accuracy. When system designers do not consider the effect of friction, the state of an UIO of [11] may not follow the actual state accurately. Hence, the residue signal calculated from UIO is also affected by the friction on the ground, and it may increase the cases of false alarm. It is desirable in practice that a detector be designed considering the friction.

The main contributions of this research are summarized as follows: (1) We propose an actuator fault detection method for a four-wheeled UGV model including a friction coefficient. Targeting the four-mecanum-wheeled UGV model of [12], we first developed actuator fault detection methods. To achieve this purpose, we used an UIO developed for linear parameter varying systems. In contrast to the approach of [11], our method can consider friction coefficients in the detector design, and it improves detection accuracy. Through simulation analysis, we demonstrated that the negative effect of terrain is reduced when friction coefficient information is used in the detectors. (2) A method of estimating friction coefficient is also proposed, since obtaining the coefficient is necessary for the detector design. The friction estimation method targeting the four-mecanum-wheeled UGV model is introduced in this paper. Existing estimation methods of [13,14,15] were not developed for the four-mecanum-wheeled UGV model. A more detailed description is shown in Section 3. (3) Finally, under various operation conditions, including UGV swarm scenarios, we evaluated the performance of the proposed method.

Here, it should be pointed out that although several fault detection methods were developed in [16,17,18,19,20], existing methods target two-wheel or four-wheel skid steered mobile robots, which are significantly different from four-mecanum-wheeled UGVs. Here, a summary of existing methods is provided. The authors of [16] developed a model-based actuator fault diagnosis method using structural analysis for a four-wheeled skid steering mobile robot. Reference [17] proposed a multiple model-based fault detection method to detect and identify actuator faults in two-wheeled mobile robots. Using a bank of Kalman filters, the fault detection was performed, and they analyzed the residue signals calculated through each filter for obtaining the accurate fault identification information. The authors of [18] presented a detection method to detect and isolate actuator and sensor faults in two-wheeled mobile robots by designing primary residual vectors which are highly sensitive to faults and less sensitive to process disturbances. Reference [19] proposed a fault detection method for two-wheeled mobile robots with parametric uncertainty. A prediction error-based fault detection algorithm was introduced that can detect wheel faults such as deformations and flat tires. The authors of [20], using extended Kalman filters, developed an actuator fault detection and isolation scheme for two-wheeled differential drive mobile robots.

The rest of this paper is organized as follows: In Section 2, we introduce a model for the four-wheeled UGV with the friction coefficient. The UIO-based actuator fault detection method and the friction coefficient estimation method are presented in Section 3. The simulation results are given in Section 4. Finally, the conclusions are formulated in Section 5.

## 2. Dynamics of Vehicles with Four Mecanum Wheels

Consider the dynamics of a UGV with four mecanum wheels [12]:

(1) $$\begin{array}{cc} \dot{x} & =Ax+B_{\rho }\tau +B_{\rho }w,\\ y & =Cx, \end{array}$$

where $x\in R^{6}$ is the vehicle state, $\tau \in R^{4}$ represents the motor torques, $w\in R^{4}$ is the motor faults, $y\in R^{4}$ represents the output signals, $A\in R^{6\times 6}$ is the system matrix, $B_{\rho }\in R^{6\times 4}$ is the input matrix, and $C\in R^{4\times 6}$ is the output matrix. The UGV motion of (1) is determined by the current vehicle state, motor torques, and motor faults. The system matrix *A* is a constant matrix determined by the friction coefficient and the mass of the vehicle. The matrix $B_{\rho }$ is a parameter varying matrix determined by the yaw angle of UGV. The output matrix *C* represents sensor information to control UGV and detect faults, which includes global position, yaw angle, and yaw rate. More specifically, the state x is given by

(2) $$\begin{array}{cc} x & ={\left[\begin{array}{cccccc} x & y & \theta & \dot{x} & \dot{y} & \dot{\theta } \end{array}\right]}^{⊤}. \end{array}$$

Here, *x* and *y* are the global positions of the UGV, and θ is the yaw angle defined as the rotation angle of UGV around the vertical axis. The motor torques τ are individually generated by the four motors, and the fault signals *w* are assumed to be additive to the torques. Since fault signal *w* is regarded as a vector added to input of each motor, a fault matrix is treated as being the same as the input matrix. The matrix *A* is given by

(3) $$\begin{array}{c} A=\left[\begin{array}{cccccc} 0 & 0 & 0 & 1 & 0 & 0\\ 0 & 0 & 0 & 0 & 1 & 0\\ 0 & 0 & 0 & 0 & 0 & 1\\ 0 & 0 & 0 & -\frac{\beta }{m} & 0 & 0\\ 0 & 0 & 0 & 0 & -\frac{\beta }{m} & 0\\ 0 & 0 & 0 & 0 & 0 & -\frac{\beta }{I} \end{array}\right], \end{array}$$

where *m* is the vehicle mass, *I* is the moment of inertia, and β is the friction coefficient. The matrix $B_{\rho }$ is represented by the linear parameter varying matrix, and it is given by

(4) $$\begin{array}{cc} B_{\rho } & =\left[\begin{array}{cccc} 0 & 0 & 0 & 0\\ 0 & 0 & 0 & 0\\ 0 & 0 & 0 & 0\\ \rho_{2} & \rho_{1} & \rho_{2} & \rho_{1}\\ \rho_{1} & -\rho_{2} & \rho_{1} & -\rho_{2}\\ h & -h & -h & h \end{array}\right]. \end{array}$$

Here, the varying parameters $\rho_{1}$ and $\rho_{2}$ depend on θ, which are given by

(5) $$\begin{array}{cc} \rho_{1} & =\frac{\cos(\theta )+\sin(\theta )}{2mR},\;\rho_{2}=\frac{\cos(\theta )-\sin(\theta )}{2mR}, \end{array}$$

and the constant *h* is defined as

(6) $$\begin{array}{c} h=\frac{a+b}{2IR}. \end{array}$$

Here, *R* is the wheel radius, 2a is the vehicle width, and 2b is the vehicle length. The matrix $B_{\rho }$ is composed of time varying parameters depending on cos(θ) and sin(θ), i.e., $\rho_{1}$ and $\rho_{2}$; and a constant determined by shape of UGV, i.e., *h*. The matrix *C* is given by

(7) $$\begin{array}{c} C=\left[\begin{array}{cccccc} 1 & 0 & 0 & 0 & 0 & 0\\ 0 & 1 & 0 & 0 & 0 & 0\\ 0 & 0 & 1 & 0 & 0 & 0\\ 0 & 0 & 0 & 0 & 0 & 1 \end{array}\right], \end{array}$$

and the state information is measured by global positioning system, inertial measurement unit, gyro sensor, etc. For easy access of parameter information, a notation table is provided in Table 1.

The assumptions of this paper are given below:

**Assumption** **1.** *A fault occurs for only one actuator at a time.*

**Assumption** **2.** *$\left|\dot{\theta }\right|\leq {\dot{\theta }}_{\max}$. Here, ${\dot{\theta }}_{\max}$ is given.*

**Assumption** **3.** *$\left|\beta \right|\leq \beta_{\max}$. Here, $\beta_{\max}$ is given.*

The assumptions imply that this paper does not consider simultaneous fault cases in actuators of the UGV, and the maximum value setting is needed to design a UIO fault detector.

## 3. Actuator Fault Detection

### 3.1. Fault Detector Design

This section introduces the actuator fault detection method for the UGV of (1). The fault detector is based on the linear parameter varying UIO introduced in [21,22], and it was designed using a nominal model given by

(8) $$\begin{array}{cc} \dot{x} & =\bar{A}x+B_{\rho }\tau +B_{\rho }^{j}w^{j}+B_{\rho }^{j^{★}}w^{j^{★}},\\ y & =Cx, \end{array}$$

where $\bar{A}$ is given by

(9) $$\begin{array}{cc} \bar{A} & =\left[\begin{array}{cccccc} 0 & 0 & 0 & 1 & 0 & 0\\ 0 & 0 & 0 & 0 & 1 & 0\\ 0 & 0 & 0 & 0 & 0 & 1\\ 0 & 0 & 0 & -\frac{\bar{\beta }}{m} & 0 & 0\\ 0 & 0 & 0 & 0 & -\frac{\bar{\beta }}{m} & 0\\ 0 & 0 & 0 & 0 & 0 & -\frac{\bar{\beta }}{I} \end{array}\right]. \end{array}$$

$\bar{\beta }$ represents the nominal friction coefficient. The fault signals are regarded as being added to individual input of each motor. For the UIO design, it needs to be separated into four columns; i.e.,

(10) $$\begin{array}{cc} B_{\rho } & =\left[\begin{array}{cccc} B_{\rho }^{1} & B_{\rho }^{2} & B_{\rho }^{3} & B_{\rho }^{4} \end{array}\right]. \end{array}$$

Here, $B_{\rho }^{j}$ denotes *j*th column matrix of $B_{\rho }$ and $B_{\rho }^{j^{★}}$ is the column matrices not including $B_{\rho }^{j}$. The fault detectors designed for each column matrix $B_{\rho }^{j}$ are given by

(11) $$\begin{array}{cc} {\dot{z}}^{j} & =N_{\rho }^{j}z^{j}+G_{\rho }^{j}\tau +L_{\rho }^{j}y,\\ {\hat{x}}^{j} & =z^{j}-H_{\rho }^{j}y,\\ {\hat{y}}^{j} & =C{\hat{x}}^{j},\\ \gamma^{j} & =\left|y-{\hat{y}}^{j}\right|,\;j=1,...,4, \end{array}$$

where $z^{j}\in R^{6}$ is the state of *j*th observer, ${\hat{x}}^{j}\in R^{6}$ is the state estimate, ${\hat{y}}^{j}\in R^{4}$ is the estimated output, and $\gamma^{j}\in R$ is the residue signal. The matrices $N_{\rho }^{j}\in R^{6\times 6}$, $G_{\rho }^{j}\in R^{6\times 4}$, $L_{\rho }^{j}\in R^{6\times 4}$, and $H_{\rho }^{j}\in R^{6\times 4}$ are the observer design matrices. For the detailed design process of the detector matrices, see Appendix A.

Now, we introduce the triggering condition of detector. The alarm is triggered if the residue $\gamma^{j}$ is larger than predefined threshold $\delta_{th}$; i.e.,

(12) $$\begin{array}{c} \gamma^{j}>\delta_{th}. \end{array}$$

Based on the triggering condition, the fault detector of (11) detects the faulty actuator. The fault detection principle is explained here. We define the estimation error $e^{j}$ and the matrix $P_{\rho }^{j}$ as $e^{j}=x-{\hat{x}}^{j}$ and $P_{\rho }^{j}=I_{6\times 6}+H_{\rho }^{j}C$, respectively. Then, $e^{j}$ is rewritten by

(13) $$\begin{array}{cc} e^{j} & =P_{\rho }^{j}x-z^{j}. \end{array}$$

The time derivative of (13) yields the following error dynamics:

(14) $$\begin{array}{cc} {\dot{e}}^{j}= & {\dot{P}}_{\rho }^{j}x+P_{\rho }^{j}\dot{x}-{\dot{z}}^{j}\\ = & {\dot{P}}_{\rho }^{j}x+P_{\rho }^{j}\left(\bar{A}x+B_{\rho }\tau +B_{\rho }^{j}w^{j}+B_{\rho }^{j^{★}}w^{j^{★}}\right)-\left(N_{\rho }^{j}z^{j}+G_{\rho }^{j}\tau +L_{\rho }^{j}y\right)\\ = & N_{\rho }^{j}e^{j}+\left({\dot{P}}_{\rho }^{j}+P_{\rho }^{j}\bar{A}-N_{\rho }^{j}P_{\rho }^{j}-L_{\rho }^{j}C\right)x+\left(P_{\rho }^{j}B_{\rho }-G_{\rho }^{j}\right)\tau +P_{\rho }^{j}B_{\rho }^{j}w^{j}+P_{\rho }^{j}B_{\rho }^{j^{★}}w^{j^{★}}. \end{array}$$

The design process of Appendix A implies

(15) $$\begin{array}{cc} {\dot{P}}_{\rho }^{j}+P_{\rho }^{j}\bar{A}-N_{\rho }^{j}P_{\rho }^{j}-L_{\rho }^{j}C & =0,\\ P_{\rho }^{j}B_{\rho }-G_{\rho }^{j} & =0,\\ P_{\rho }^{j}B_{\rho }^{j} & =0. \end{array}$$

Then, the equation is briefly summarized as

(16) $$\begin{array}{cc} {\dot{e}}^{j} & =N_{\rho }^{j}e^{j}+P_{\rho }^{j}B_{\rho }^{j^{★}}w^{j^{★}}, \end{array}$$

where $N_{\rho }^{j}$ is Hurwitz.

The detector ${\hat{x}}^{j}$ estimates the vehicle state x, even if the unknown fault signal $w^{j}$ occurs in the *j*th actuator. Recall that, by Assumption 1, no fault occurs in other actuators except for *j*th. However, if $w^{j^{★}}$ occurs, the error dynamics of the *j*th detector does not converge to zero due to the term of $P_{\rho }^{j}B_{\rho }^{j^{★}}w^{j^{★}}$. This implies that the residue signals except for $\gamma^{j}$ are affected by the fault. This principle provides the important clue for the fault isolation, and the residue results for faulty actuator are summarized in Table 2. Here, T is true and F is false.

It should be noted that when *C* is a 6 by 6 identity matrix, we design conditions for the UIO treating three inputs as unknown. This, if successful, will give a detector that responds to only a single input. Thus, designing such detector for each one of the four inputs will yield four detectors that individually detect a fault for each of the four actuators. Then, Assumption 1 can be removed. However, since $P_{\rho }^{j}B_{\rho }^{j^{★}}$ becomes a zero matrix in this case, no response to the residue signal appears and the detector may not detect the faults. Consequently, the detector presented in this work appears to be the best that can be done.

### 3.2. Discussion on Friction Coefficient Estimation

**Figure 1:**
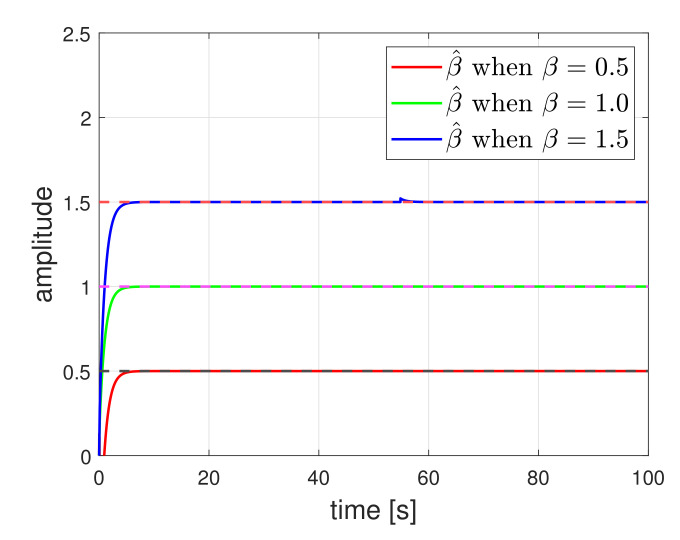
Friction coefficient estimation.

Proposing a friction estimation method may be worthy work. The estimation method using the sensor signals is here introduced. The estimation algorithm comes from previous dynamics—i.e.,

(17) $$\begin{array}{c} \hat{\beta }=\frac{I}{\dot{\theta }}\times \left[D\left(\dot{\theta }\right)+h\tau_{1}-h\tau_{2}-h\tau_{3}+h\tau_{4}\right], \end{array}$$

where $\hat{\beta }$ denotes the estimated friction, and D(·) is a differential filter. Now, we evaluate the performance of the proposed estimation method when a vehicle tracks a circle. The simulation parameters were same as those in the following section and the estimation results are displayed in Figure 1. We present three estimation results for β=0.5, β=1.0, and β=1.5. The estimates $\hat{\beta }$ closely estimate the actual frictions β. The estimate results may be usefully employed in the detector design.

Here, it is worth noting that friction coefficient estimation method for four-wheel mecanum UGV was developed for this study. Although existing friction estimation methods do not target four-mecanum-wheeled UGVs, introducing existing approaches may be worthy work. Reference [13] developed reliable estimation algorithms for independent friction coefficients at each individual wheel of the vehicle. Three observers using engine torque, brake torque, and GPS measurements were employed for estimating slip ratios and longitudinal tire forces, and the friction coefficients were identified using a recursive least-squares method. The authors of [14], targeting the steering vehicular dynamics, proposed a method capable of estimating the tire road friction coefficient using conventional Kalman filter and recursive least-squares method. Reference [15] proposed a real-time estimation method for maximum friction coefficient and optimal slip ratio for securing the maneuverability of mobile robots with rubber tires.

When existing results of [13,14,15] are utilized, we may develop fault detectors with higher accuracy than our estimation method. The performance improvement may occur through comparative analysis with existing methods and new research results may be obtained. However, instead of developing a more sophisticated estimation technique, this paper focuses on how friction estimates can be used when the friction estimate is given. We show that when an estimate is given, the fault detector can have higher accuracy compared to existing methods not using friction information.

## 4. Simulation Results

### 4.1. Actuator Fault Detection of Individual UGV

The performance of proposed detector is evaluated here. The parameter values used in simulation are summarized in Table 3.

**Figure 2:**
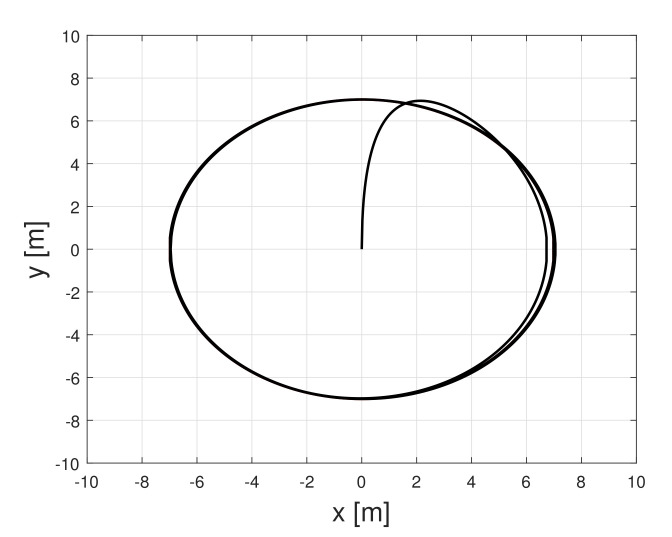
UGV circle trajectory result.

To control UGV, a flatness based controller of [12] was employed, which is briefly introduced in Appendix A. The purpose of this control is to track a circle reference, and the operation result is shown in Figure 2.

**Figure 3:**
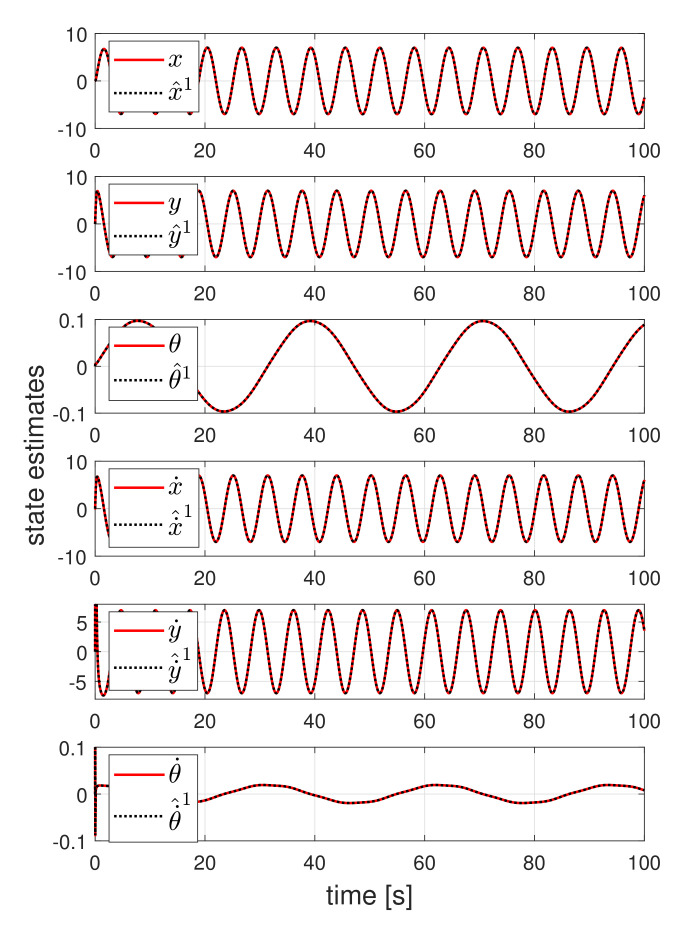
State estimates of the UIO designed for first actuator from when the UGV tracked the circle reference.

Now, we analyze the detector design result. Figure 3 shows the state estimates of the first detector when the UGV tracked the circle reference. To observe the estimation performance, the actuator fault is not considered in this simulation. All state estimates asymptotically follow the actual UGV state, even if the UGV is operated under the existence of friction. As the effect of friction is considered in the detector design process, the detector can estimate the actual state.

**Figure 4:**
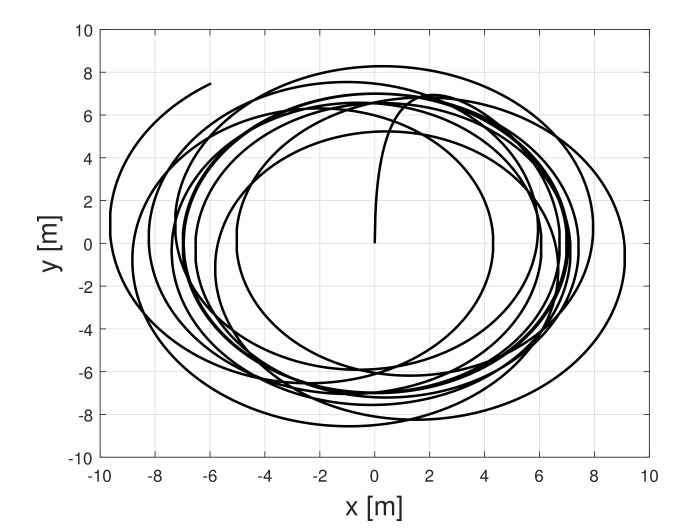
UGV circle trajectory with an actuator fault.

**Figure 5:**
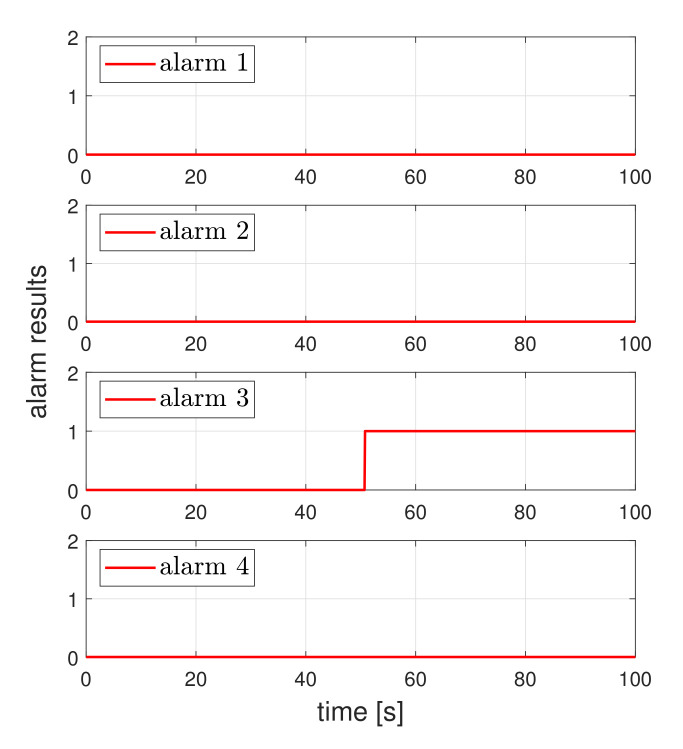
Actuator fault detection result for the circle tracking case. A value of one indicates an actuator fault and the value of zero indicates normal operation.

Next, the effectiveness of the detector is evaluated. Consider the scenario when a UGV tracks a circle reference and a third actuator fault occurs at about 50 s. Figure 4 shows the tracking result of UGV under the actuator fault. As expected, UGV loses the tracking performance. Figure 5 shows the fault detection result under the circle tracking operation. The proposed method achieved the actuator fault detection and isolation, even if the UGV was affected by the existence of friction.

**Figure 6:**
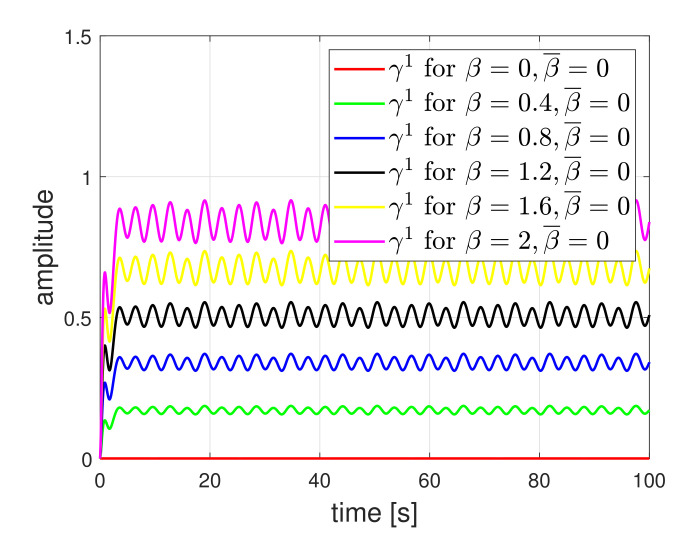
Residue signal of first detector when friction is not considered in the detector design.

**Figure 7:**
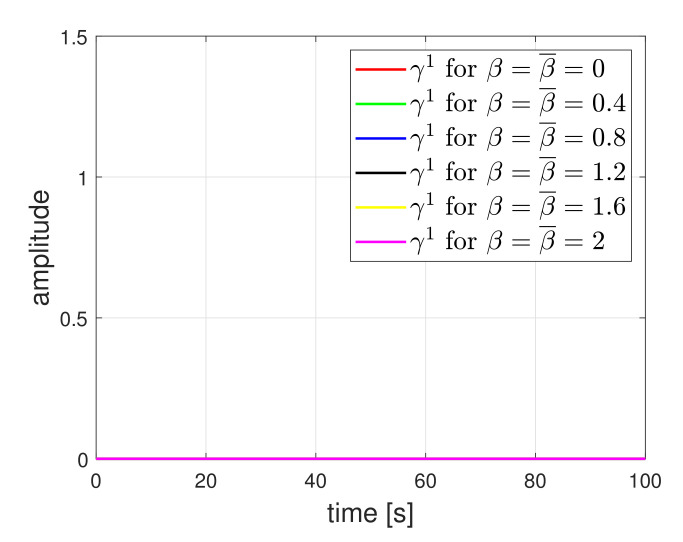
Residue signal of first detector when friction is considered in the detector design.

**Figure 8:**
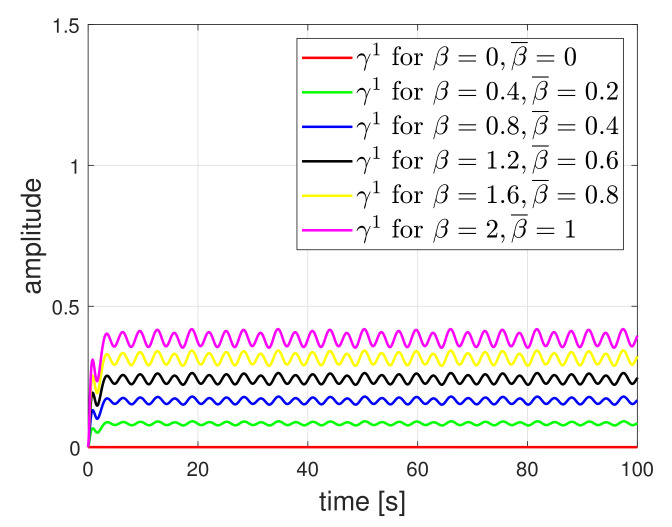
Residue signal of first detector when friction is considered in the detector design, but it is not accurate.

For comparison, we show the residue results of first detector under the circle tracking scenario where the friction coefficient is not considered in the detector design. The residue results are plotted in Figure 6. The residues were affected by the friction. As the amplitude of friction was larger, the effect on the residue also increased. However, as shown in Figure 7, when the friction was considered in the design, the effect was clearly reduced. Nevertheless, if the actual friction value is not accurately known, i.e., the nominal value differs from the actual value, the effect of friction will still appear in the residue. The results are shown in Figure 8. Selecting $\bar{\beta }$ through several experiment procedures may be necessary work. Here, it needs to be emphasized that the results of Figure 6 represent the existing detection methods of [11]. When the friction coefficient is considered in the detector design, the detection accuracy can be improved. Hence, it implies that our method is practically necessary for obtaining high accuracy.

### 4.2. Performance Evaluation under Swarm Scenario

**Figure 9:**
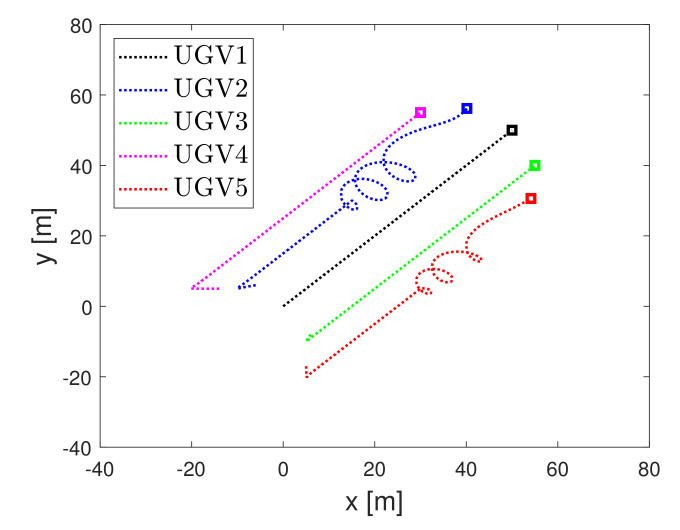
Swarm operation of UGV.

**Figure 10:**
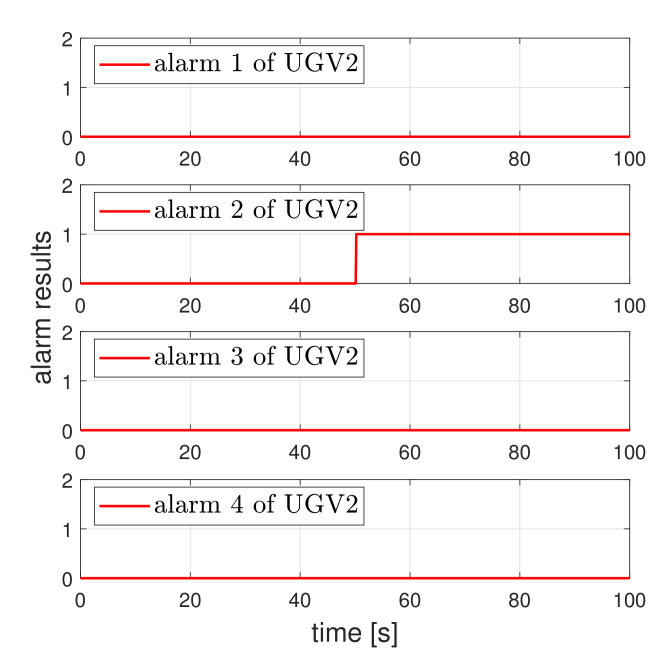
Fault isolation of UGV2. A value of one indicates an actuator fault and the value of zero indicates normal operation.

**Figure 11:**
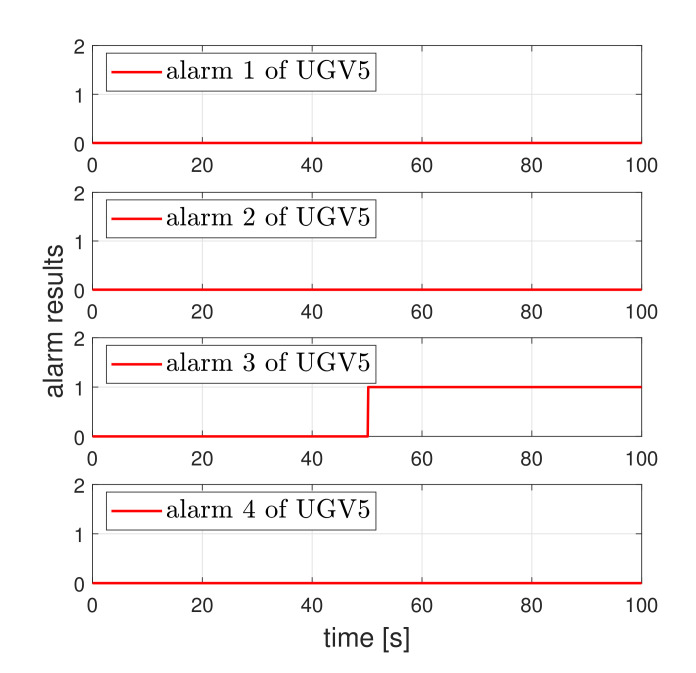
Fault isolation for UGV5. A value of one indicates an actuator fault and the value of zero indicates normal operation.

Now, we show that the proposed detector can isolate faulty actuator of the faulty UGV under the swarm scenario. For this, the fault detectors needed to be designed for all UGVs. Consider the swarm scenario where five UGVs are controlled as a triangle formation. We assumed that the faults in the second actuator of second UGV and the third actuator of fifth UGV occurred at 50 s.The operation result is shown in Figure 9. Due to the actuator fault, the second vehicle and fifth vehicles deviated from the formation. Figure 10 and Figure 11 show that the proposed detector isolated the faulty actuator under the swarm scenario. This shows that our method may be applied to the swarmed UGV and the individual UGV.

## 5. Conclusions

We developed an actuator fault detection method for four-mecanum-wheeled unmanned ground vehicle dynamics with friction coefficients. The proposed actuator fault detection method uses unknown input observers developed for linear parameter varying systems. Since the friction coefficients were used in the design of the proposed detector, we additionally proposed a method of friction coefficient estimation for mecanum-wheel UGV dynamics. The obtained simulation results qualitatively show that detection performance of the proposed method is superior to that of the existing approach which does not take friction coefficients into account. The effect of friction estimation error on the accuracy of detection is quantified by simulating the system for many cases. Smaller estimation errors result in higher detection accuracy. Through various simulation results, we showed the effectiveness of the proposed fault detection method. This approach is applicable to swarm systems and individual UGVs. Future work will include investigation of the effect on the detector performance by extending other friction estimation methods available in the literature to mecanum wheels, and extending the detection capability to the faults that are occurring simultaneously. 

## Figures and Tables

**Table 1:** UGV parameters.

vehicle state	x	friction coefficient	β
motor torques	τ	varying parameters	$\rho_{1}$, $\rho_{2}$
motor fault signals	*w*	UGV constant	*h*
global position	*x*, *y*	wheel radius	*R*
yaw angle	θ	vehicle width	2a
vehicle mass	*m*	vehicle length	2b
yaw moment of inertia	*I*	gravity acceleration	*g*

**Table 2:** Residue results for faulty actuator.

	$\gamma^{1}>\delta_{\mathrm{th}}$	$\gamma^{2}>\delta_{\mathrm{th}}$	$\gamma^{3}>\delta_{\mathrm{th}}$	$\gamma^{4}>\delta_{\mathrm{th}}$
$w^{1}\neq 0$	F	T	T	T
$w^{2}\neq 0$	T	F	T	T
$w^{3}\neq 0$	T	T	F	T
$w^{4}\neq 0$	T	T	T	F

**Table 3:** Parameter information used in the simulation.

vehicle mass	*m*	6
yaw moment of inertia	*I*	0.0945
wheel radius	*R*	0.05
vehicle width	2a	0.22
vehicle length	2b	0.36
acceleration due to gravity	*g*	9.8
friction coefficient	β	1.2
nominal friction coefficient	$\bar{\beta }$	1.2
threshold value	$\delta_{th}$	0.5
maximum of yaw rate	${\dot{\theta }}_{\max}$	3
maximum of friction coefficient	$\beta_{\max}$	2
proportional control gain	$K_{P,i}$	[6.2;8.4;25]
integral control gain	$K_{I,i}$	[0.4;0.6;0.2]
derivative control gain	$K_{D,i}$	[8;7;418]

## Data Availability

Authors will be able to provide simulation code and necessary data if there is no confidential issue.

## Appendix A

**Fault detector design:** We below introduce fault detector design method of total six steps.

Step 1: Calculate $H_{\rho }^{j}=-B_{\rho }^{j}{\left(CB_{\rho }^{j}\right)}^{†}$. Then,

$$H_{\rho }^{1}=\left[\begin{array}{cccc} 0 & 0 & 0 & 0\\ 0 & 0 & 0 & 0\\ 0 & 0 & 0 & 0\\ 0 & 0 & 0 & -\frac{\rho_{2}}{h}\\ 0 & 0 & 0 & -\frac{\rho_{1}}{h}\\ 0 & 0 & 0 & -1 \end{array}\right],\;H_{\rho }^{2}=\left[\begin{array}{cccc} 0 & 0 & 0 & 0\\ 0 & 0 & 0 & 0\\ 0 & 0 & 0 & 0\\ 0 & 0 & 0 & \frac{\rho_{1}}{h}\\ 0 & 0 & 0 & -\frac{\rho_{2}}{h}\\ 0 & 0 & 0 & -1 \end{array}\right],$$

$$H_{\rho }^{3}=\left[\begin{array}{cccc} 0 & 0 & 0 & 0\\ 0 & 0 & 0 & 0\\ 0 & 0 & 0 & 0\\ 0 & 0 & 0 & \frac{\rho_{2}}{h}\\ 0 & 0 & 0 & \frac{\rho_{1}}{h}\\ 0 & 0 & 0 & -1 \end{array}\right],\;H_{\rho }^{4}=\left[\begin{array}{cccc} 0 & 0 & 0 & 0\\ 0 & 0 & 0 & 0\\ 0 & 0 & 0 & 0\\ 0 & 0 & 0 & -\frac{\rho_{1}}{h}\\ 0 & 0 & 0 & \frac{\rho_{2}}{h}\\ 0 & 0 & 0 & -1 \end{array}\right].$$

Step 2: Calculate $P_{\rho }^{j}=I_{6\times 6}+H_{\rho }^{j}C$. Then,

$$P_{\rho }^{1}=\left[\begin{array}{cccccc} 1 & 0 & 0 & 0 & 0 & 0\\ 0 & 1 & 0 & 0 & 0 & 0\\ 0 & 0 & 1 & 0 & 0 & 0\\ 0 & 0 & 0 & 1 & 0 & -\frac{\rho_{2}}{h}\\ 0 & 0 & 0 & 0 & 1 & -\frac{\rho_{1}}{h}\\ 0 & 0 & 0 & 0 & 0 & 0 \end{array}\right],\;P_{\rho }^{2}=\left[\begin{array}{cccccc} 1 & 0 & 0 & 0 & 0 & 0\\ 0 & 1 & 0 & 0 & 0 & 0\\ 0 & 0 & 1 & 0 & 0 & 0\\ 0 & 0 & 0 & 1 & 0 & \frac{\rho_{1}}{h}\\ 0 & 0 & 0 & 0 & 1 & -\frac{\rho_{2}}{h}\\ 0 & 0 & 0 & 0 & 0 & 0 \end{array}\right],$$

$$P_{\rho }^{3}=\left[\begin{array}{cccccc} 1 & 0 & 0 & 0 & 0 & 0\\ 0 & 1 & 0 & 0 & 0 & 0\\ 0 & 0 & 1 & 0 & 0 & 0\\ 0 & 0 & 0 & 1 & 0 & \frac{\rho_{2}}{h}\\ 0 & 0 & 0 & 0 & 1 & \frac{\rho_{1}}{h}\\ 0 & 0 & 0 & 0 & 0 & 0 \end{array}\right],\;P_{\rho }^{4}=\left[\begin{array}{cccccc} 1 & 0 & 0 & 0 & 0 & 0\\ 0 & 1 & 0 & 0 & 0 & 0\\ 0 & 0 & 1 & 0 & 0 & 0\\ 0 & 0 & 0 & 1 & 0 & -\frac{\rho_{1}}{h}\\ 0 & 0 & 0 & 0 & 1 & \frac{\rho_{2}}{h}\\ 0 & 0 & 0 & 0 & 0 & 0 \end{array}\right].$$

Step 3: Calculate $G_{\rho }^{j}=P_{\rho }^{j}B_{\rho }$. Then,

$$G_{\rho }^{1}=\left[\begin{array}{cccc} 0 & 0 & 0 & 0\\ 0 & 0 & 0 & 0\\ 0 & 0 & 0 & 0\\ 0 & \rho_{1}+\rho_{2} & 2\rho_{2} & \rho_{1}-\rho_{2}\\ 0 & \rho_{1}-\rho_{2} & 2\rho_{1} & -\rho_{1}-\rho_{2}\\ 0 & 0 & 0 & 0 \end{array}\right],\;G_{\rho }^{2}=\left[\begin{array}{cccc} 0 & 0 & 0 & 0\\ 0 & 0 & 0 & 0\\ 0 & 0 & 0 & 0\\ \rho_{1}+\rho_{2} & 0 & \rho_{2}-\rho_{1} & 2\rho_{1}\\ \rho_{1}-\rho_{2} & 0 & \rho_{1}+\rho_{2} & -2\rho_{2}\\ 0 & 0 & 0 & 0 \end{array}\right],$$

$$G_{\rho }^{3}=\left[\begin{array}{cccc} 0 & 0 & 0 & 0\\ 0 & 0 & 0 & 0\\ 0 & 0 & 0 & 0\\ 2\rho_{2} & \rho_{1}-\rho_{2} & 0 & \rho_{1}+\rho_{2}\\ 2\rho_{1} & -\rho_{1}-\rho_{2} & 0 & \rho_{1}-\rho_{2}\\ 0 & 0 & 0 & 0 \end{array}\right],\;G_{\rho }^{4}=\left[\begin{array}{cccc} 0 & 0 & 0 & 0\\ 0 & 0 & 0 & 0\\ 0 & 0 & 0 & 0\\ \rho_{2}-\rho_{1} & 2\rho_{1} & \rho_{1}+\rho_{2} & 0\\ \rho_{1}+\rho_{2} & -2\rho_{2} & \rho_{1}-\rho_{2} & 0\\ 0 & 0 & 0 & 0 \end{array}\right].$$

Step 4: Calculate ${\tilde{A}}_{\rho ,\dot{\rho }}^{j}={\dot{P}}_{\rho }^{j}+P_{\rho }^{j}A$. First, we calculate ${\dot{P}}_{\rho }^{1}$, i.e.,

$${\dot{P}}_{\rho }^{1}=\left[\begin{array}{cccccc} 0 & 0 & 0 & 0 & 0 & 0\\ 0 & 0 & 0 & 0 & 0 & 0\\ 0 & 0 & 0 & 0 & 0 & 0\\ 0 & 0 & 0 & 0 & 0 & -\frac{{\dot{\rho }}_{2}}{h}\\ 0 & 0 & 0 & 0 & 0 & -\frac{{\dot{\rho }}_{1}}{h}\\ 0 & 0 & 0 & 0 & 0 & 0 \end{array}\right].$$

From ${\dot{\rho }}_{1}=-\rho_{2}\dot{\theta }$ and ${\dot{\rho }}_{2}=-\rho_{1}\dot{\theta }$, we have

$$\begin{array}{cc} {\dot{P}}_{\rho }^{1} & =\left[\begin{array}{cccccc} 0 & 0 & 0 & 0 & 0 & 0\\ 0 & 0 & 0 & 0 & 0 & 0\\ 0 & 0 & 0 & 0 & 0 & 0\\ 0 & 0 & 0 & 0 & 0 & \frac{\rho_{1}\dot{\theta }}{h}\\ 0 & 0 & 0 & 0 & 0 & \frac{\rho_{2}\dot{\theta }}{h}\\ 0 & 0 & 0 & 0 & 0 & 0 \end{array}\right]. \end{array}$$

Then, it is given by

$$\begin{array}{cc} {\tilde{A}}_{\rho ,\dot{\rho }}^{1} & =\left[\begin{array}{cccccc} 0 & 0 & 0 & 1 & 0 & 0\\ 0 & 0 & 0 & 0 & 1 & 0\\ 0 & 0 & 0 & 0 & 0 & 1\\ 0 & 0 & 0 & -\frac{\bar{\beta }}{m} & 0 & \frac{\rho_{1}\dot{\theta }}{h}+\frac{\rho_{2}\bar{\beta }}{hI}\\ 0 & 0 & 0 & 0 & -\frac{\bar{\beta }}{m} & \frac{\rho_{2}\dot{\theta }}{h}+\frac{\rho_{1}\bar{\beta }}{hI}\\ 0 & 0 & 0 & 0 & 0 & 0 \end{array}\right]. \end{array}$$

The matrices ${\tilde{A}}_{\rho ,\dot{\rho }}^{2}$, ${\tilde{A}}_{\rho ,\dot{\rho }}^{3}$, and ${\tilde{A}}_{\rho ,\dot{\rho }}^{4}$ are similarly obtained as

$$\begin{array}{cc} {\tilde{A}}_{\rho ,\dot{\rho }}^{2} & =\left[\begin{array}{cccccc} 0 & 0 & 0 & 1 & 0 & 0\\ 0 & 0 & 0 & 0 & 1 & 0\\ 0 & 0 & 0 & 0 & 0 & 1\\ 0 & 0 & 0 & -\frac{\bar{\beta }}{m} & 0 & -\frac{\rho_{2}\dot{\theta }}{h}-\frac{\rho_{1}\bar{\beta }}{hI}\\ 0 & 0 & 0 & 0 & -\frac{\bar{\beta }}{m} & \frac{\rho_{1}\dot{\theta }}{h}+\frac{\rho_{2}\bar{\beta }}{hI}\\ 0 & 0 & 0 & 0 & 0 & 0 \end{array}\right], \end{array}$$

$$\begin{array}{cc} {\tilde{A}}_{\rho ,\dot{\rho }}^{3} & =\left[\begin{array}{cccccc} 0 & 0 & 0 & 1 & 0 & 0\\ 0 & 0 & 0 & 0 & 1 & 0\\ 0 & 0 & 0 & 0 & 0 & 1\\ 0 & 0 & 0 & -\frac{\bar{\beta }}{m} & 0 & -\frac{\rho_{1}\dot{\theta }}{h}-\frac{\rho_{2}\bar{\beta }}{hI}\\ 0 & 0 & 0 & 0 & -\frac{\bar{\beta }}{m} & -\frac{\rho_{2}\dot{\theta }}{h}-\frac{\rho_{1}\bar{\beta }}{hI}\\ 0 & 0 & 0 & 0 & 0 & 0 \end{array}\right], \end{array}$$

$$\begin{array}{cc} {\tilde{A}}_{\rho ,\dot{\rho }}^{4} & =\left[\begin{array}{cccccc} 0 & 0 & 0 & 1 & 0 & 0\\ 0 & 0 & 0 & 0 & 1 & 0\\ 0 & 0 & 0 & 0 & 0 & 1\\ 0 & 0 & 0 & -\frac{\bar{\beta }}{m} & 0 & \frac{\rho_{2}\dot{\theta }}{h}+\frac{\rho_{1}\bar{\beta }}{hI}\\ 0 & 0 & 0 & 0 & -\frac{\bar{\beta }}{m} & -\frac{\rho_{1}\dot{\theta }}{h}-\frac{\rho_{2}\bar{\beta }}{hI}\\ 0 & 0 & 0 & 0 & 0 & 0 \end{array}\right]. \end{array}$$

Step 5: Calculate $N_{\rho }^{j}={\tilde{A}}_{\rho ,\dot{\rho }}^{j}-K_{\mu }^{j}C$. First, define $s_{1}^{1}$ and $s_{2}^{1}$ as

$$\begin{array}{cc} s_{1}^{1} & =\frac{\rho_{1}\dot{\theta }}{h}+\frac{\rho_{2}\bar{\beta }}{hI}\in \left[s_{1,\min},\;s_{1,\max}\right],\\ s_{2}^{1} & =\frac{\rho_{2}\dot{\theta }}{h}+\frac{\rho_{1}\bar{\beta }}{hI}\in \left[s_{2,\min},\;s_{2,\max}\right]. \end{array}$$

Here, $s_{1,\min}$, $s_{1,\max}$, $s_{2,\min}$, and $s_{2,\max}$ are defined by

$$\begin{array}{cc} s_{1,\min} & =\left(\frac{{\dot{\theta }}_{\max}}{h}+\frac{\beta_{\max}}{hI}\right)\times \frac{-2}{2mR},\;s_{1,\max}=\left(\frac{{\dot{\theta }}_{\max}}{h}+\frac{\beta_{\max}}{hI}\right)\times \frac{2}{2mR},\\ s_{2,\min} & =\left(\frac{{\dot{\theta }}_{\max}}{h}+\frac{\beta_{\max}}{hI}\right)\times \frac{-2}{2mR},\;s_{2,\max}=\left(\frac{{\dot{\theta }}_{\max}}{h}+\frac{\beta_{\max}}{hI}\right)\times \frac{2}{2mR}. \end{array}$$

Then, ${\tilde{A}}_{\rho ,\dot{\rho }}^{1}$ is expressed by

$$\begin{array}{cc} {\tilde{A}}_{\rho ,\dot{\rho }}^{1} & =\left[\begin{array}{cccccc} 0 & 0 & 0 & 1 & 0 & 0\\ 0 & 0 & 0 & 0 & 1 & 0\\ 0 & 0 & 0 & 0 & 0 & 1\\ 0 & 0 & 0 & -\frac{\bar{\beta }}{m} & 0 & s_{1}^{1}\\ 0 & 0 & 0 & 0 & -\frac{\bar{\beta }}{m} & s_{2}^{1}\\ 0 & 0 & 0 & 0 & 0 & 0 \end{array}\right]. \end{array}$$

Define

$$\begin{array}{cc} g_{1}^{1} & =\frac{s_{1}^{1}-s_{1,\min}}{s_{1,\max}-s_{1,\min}},\;g_{2}^{1}=\frac{s_{1,\max}-s_{1}^{1}}{s_{1,\max}-s_{1,\min}},\\ g_{3}^{1} & =\frac{s_{2}^{1}-s_{2,\min}}{s_{2,\max}-s_{2,\min}},\;g_{4}^{1}=\frac{s_{2,\max}-s_{2}^{1}}{s_{2,\max}-s_{2,\min}}. \end{array}$$

Note that $s_{1}^{1}$ and $s_{2}^{1}$ can be represented as

$$\begin{array}{cc} s_{1}^{1} & =s_{1,\max}\;g_{1}^{1}+s_{1,\min}\;g_{2}^{1},\\ s_{2}^{1} & =s_{2,\max}\;g_{3}^{1}+s_{2,\min}\;g_{4}^{1}. \end{array}$$

From following facts, i.e.,

$$\begin{array}{cc} g_{1}^{1}+g_{2}^{1} & =1,\;g_{3}^{1}+g_{4}^{1}=1, \end{array}$$

we have

$$\begin{array}{cc} {\tilde{A}}_{\rho ,\dot{\rho }}^{1} & =\mu_{1}^{1}{\tilde{A}}_{1}+\mu_{2}^{1}{\tilde{A}}_{2}+\mu_{3}^{1}{\tilde{A}}_{3}+\mu_{4}^{1}{\tilde{A}}_{4}. \end{array}$$

Here, $\mu_{1}^{1}=g_{1}^{1}g_{3}^{1}$, $\mu_{2}^{1}=g_{1}^{1}g_{4}^{1}$, $\mu_{3}^{1}=g_{2}^{1}g_{3}^{1}$, $\mu_{4}^{1}=g_{2}^{1}g_{4}^{1}$, and

$${\tilde{A}}_{1}=\left[\begin{array}{cccccc} 0 & 0 & 0 & 1 & 0 & 0\\ 0 & 0 & 0 & 0 & 1 & 0\\ 0 & 0 & 0 & 0 & 0 & 1\\ 0 & 0 & 0 & -\frac{\bar{\beta }}{m} & 0 & s_{1,\max}\\ 0 & 0 & 0 & 0 & -\frac{\bar{\beta }}{m} & s_{2,\max}\\ 0 & 0 & 0 & 0 & 0 & 0 \end{array}\right],\;{\tilde{A}}_{2}=\left[\begin{array}{cccccc} 0 & 0 & 0 & 1 & 0 & 0\\ 0 & 0 & 0 & 0 & 1 & 0\\ 0 & 0 & 0 & 0 & 0 & 1\\ 0 & 0 & 0 & -\frac{\bar{\beta }}{m} & 0 & s_{1,\max}\\ 0 & 0 & 0 & 0 & -\frac{\bar{\beta }}{m} & s_{2,\min}\\ 0 & 0 & 0 & 0 & 0 & 0 \end{array}\right],$$

$${\tilde{A}}_{3}=\left[\begin{array}{cccccc} 0 & 0 & 0 & 1 & 0 & 0\\ 0 & 0 & 0 & 0 & 1 & 0\\ 0 & 0 & 0 & 0 & 0 & 1\\ 0 & 0 & 0 & -\frac{\bar{\beta }}{m} & 0 & s_{1,\min}\\ 0 & 0 & 0 & 0 & -\frac{\bar{\beta }}{m} & s_{2,\max}\\ 0 & 0 & 0 & 0 & 0 & 0 \end{array}\right],\;{\tilde{A}}_{4}=\left[\begin{array}{cccccc} 0 & 0 & 0 & 1 & 0 & 0\\ 0 & 0 & 0 & 0 & 1 & 0\\ 0 & 0 & 0 & 0 & 0 & 1\\ 0 & 0 & 0 & -\frac{\bar{\beta }}{m} & 0 & s_{1,\min}\\ 0 & 0 & 0 & 0 & -\frac{\bar{\beta }}{m} & s_{2,\min}\\ 0 & 0 & 0 & 0 & 0 & 0 \end{array}\right].$$

Similarly, ${\tilde{A}}_{\rho ,\dot{\rho }}^{2}$, ${\tilde{A}}_{\rho ,\dot{\rho }}^{3}$, and ${\tilde{A}}_{\rho ,\dot{\rho }}^{4}$ can be obtained as

$$\begin{array}{cc} {\tilde{A}}_{\rho ,\dot{\rho }}^{2} & =\mu_{1}^{2}{\tilde{A}}_{1}+\mu_{2}^{2}{\tilde{A}}_{2}+\mu_{3}^{2}{\tilde{A}}_{3}+\mu_{4}^{2}{\tilde{A}}_{4},\\ {\tilde{A}}_{\rho ,\dot{\rho }}^{3} & =\mu_{1}^{3}{\tilde{A}}_{1}+\mu_{2}^{3}{\tilde{A}}_{2}+\mu_{3}^{3}{\tilde{A}}_{3}+\mu_{4}^{3}{\tilde{A}}_{4},\\ {\tilde{A}}_{\rho ,\dot{\rho }}^{4} & =\mu_{1}^{4}{\tilde{A}}_{1}+\mu_{2}^{4}{\tilde{A}}_{2}+\mu_{3}^{4}{\tilde{A}}_{3}+\mu_{4}^{4}{\tilde{A}}_{4}. \end{array}$$

Step 6: Calculate $L_{\rho }^{j}=K_{\mu }^{j}-N_{\rho }^{j}H_{\rho }^{j}$. Here, $K_{\mu }^{j}$ is designed such that $N_{\rho }^{j}$ is stable.

**Tracking Controller Used in Simulation:** For designing controller, we refer to [12]. Define

$$\begin{array}{cc} \varepsilon_{1} & =x_{d}-x,\;\varepsilon_{2}=y_{d}-y,\;\varepsilon_{3}=\theta_{d}-\theta . \end{array}$$

Here, $x_{d}$, $y_{d}$, and $\theta_{d}$ are desired references. Also, define

$$\begin{array}{cc} \xi = & \left[\begin{array}{c} {\ddot{x}}_{d}\\ {\ddot{y}}_{d}\\ {\ddot{\theta }}_{d} \end{array}\right]+\left[\begin{array}{ccc} K_{P,1} & 0 & 0\\ 0 & K_{P,2} & 0\\ h & 0 & K_{P,3} \end{array}\right]\left[\begin{array}{c} \varepsilon_{1}\\ \varepsilon_{2}\\ \varepsilon_{3} \end{array}\right]\\ & +\left[\begin{array}{ccc} K_{I,1} & 0 & 0\\ 0 & K_{I,2} & 0\\ h & 0 & K_{I,3} \end{array}\right]\left[\begin{array}{c} \int \varepsilon_{1}\\ \int \varepsilon_{2}\\ \int \varepsilon_{3} \end{array}\right]+\left[\begin{array}{ccc} K_{D,1} & 0 & 0\\ 0 & K_{D,2} & 0\\ h & 0 & K_{D,3} \end{array}\right]\left[\begin{array}{c} {\dot{\varepsilon }}_{1}\\ {\dot{\varepsilon }}_{2}\\ {\dot{\varepsilon }}_{3} \end{array}\right], \end{array}$$

where $K_{P,i}$ is proportional gain, $K_{I,i}$ is integral gain, and $K_{D,i}$ is derivative gain. To control UGV, τ is designed as

$$\begin{array}{cc} \tau = & {\left[\begin{array}{cccc} \frac{1}{2mR} & 0 & \frac{1}{2mR} & 0\\ 0 & \frac{1}{2mR} & 0 & \frac{1}{2mR}\\ h & -h & -h & h \end{array}\right]}^{†}\times \{\left[\begin{array}{ccc} \frac{\cos(\theta )-\sin(\theta )}{2} & \frac{\cos(\theta )+\sin(\theta )}{2} & 0\\ \frac{\cos(\theta )+\sin(\theta )}{2} & \frac{\sin(\theta )-\cos(\theta )}{2} & 0\\ 0 & 0 & 1 \end{array}\right]\xi \\ & +\left[\begin{array}{ccc} \frac{\bar{\beta }\left(\cos\left(\theta \right)-\sin\left(\theta \right)\right)}{2m} & \frac{\bar{\beta }\left(\cos\left(\theta \right)+\sin\left(\theta \right)\right)}{2m} & 0\\ \frac{\bar{\beta }\left(\cos\left(\theta \right)+\sin\left(\theta \right)\right)}{2m} & \frac{\bar{\beta }\left(\sin\left(\theta \right)-\cos\left(\theta \right)\right)}{2m} & 0\\ 0 & 0 & \frac{\bar{\beta }}{I} \end{array}\right]\left[\begin{array}{c} \dot{x}\\ \dot{y}\\ \dot{\theta } \end{array}\right]\}. \end{array}$$

The tracking controller is completed.
